# Supplementary figures and images for: A WDR35-dependent coat protein complex transports ciliary membrane cargo vesicles to cilia
Source: eLife. 2021 Nov 4;10:e69786. doi: 10.7554/eLife.69786 (PMC8754431; doi:10.7554/eLife.69786)

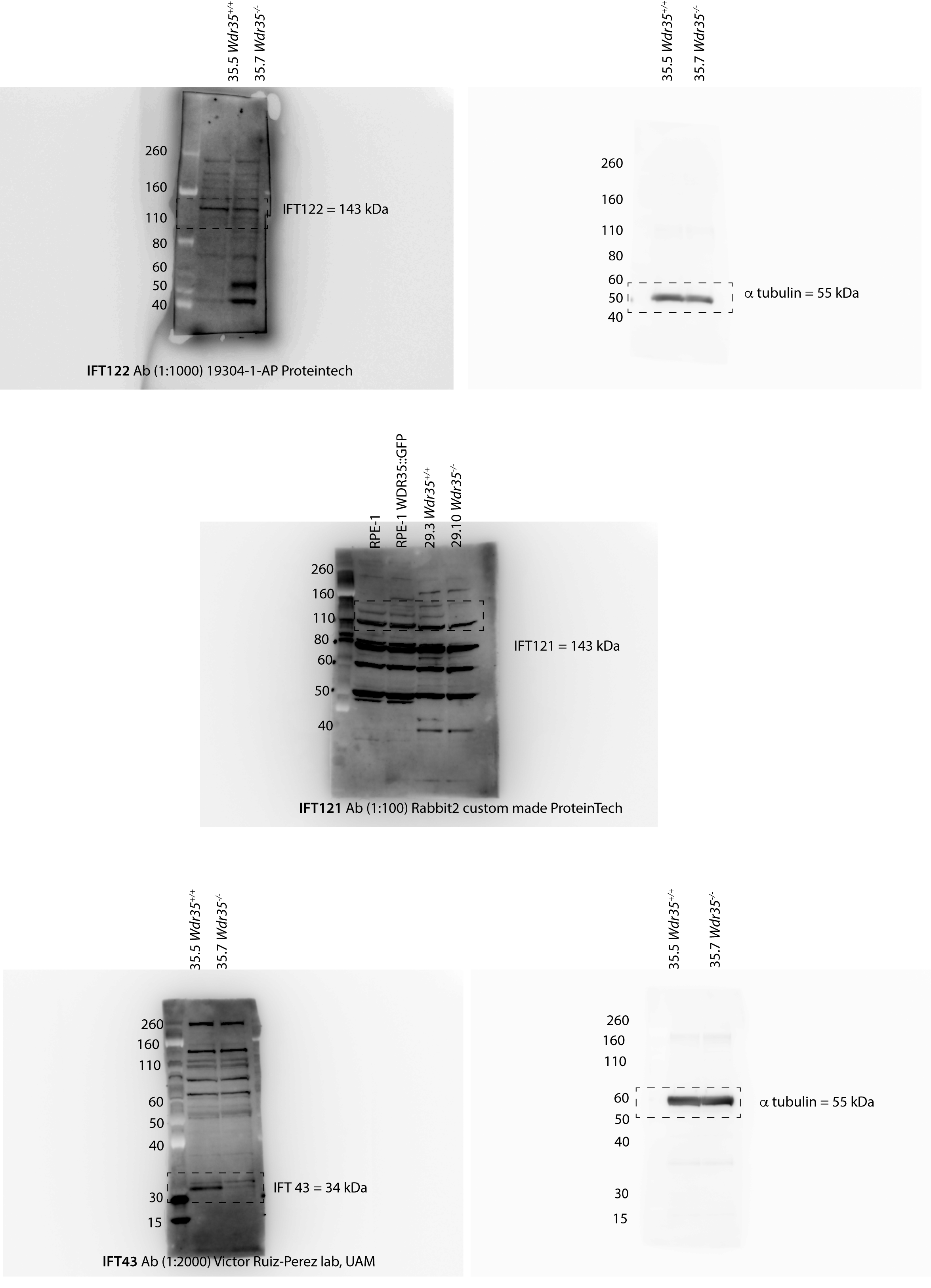

Supplement: Figure 3—source data 1. [file elife-69786-fig3-data1.zip › Figure 3 Source blots upload AUG 2021/Figure3C/Figure 3C Source labelled blots /Figure 3C labelled IFT122 121 43.tif]

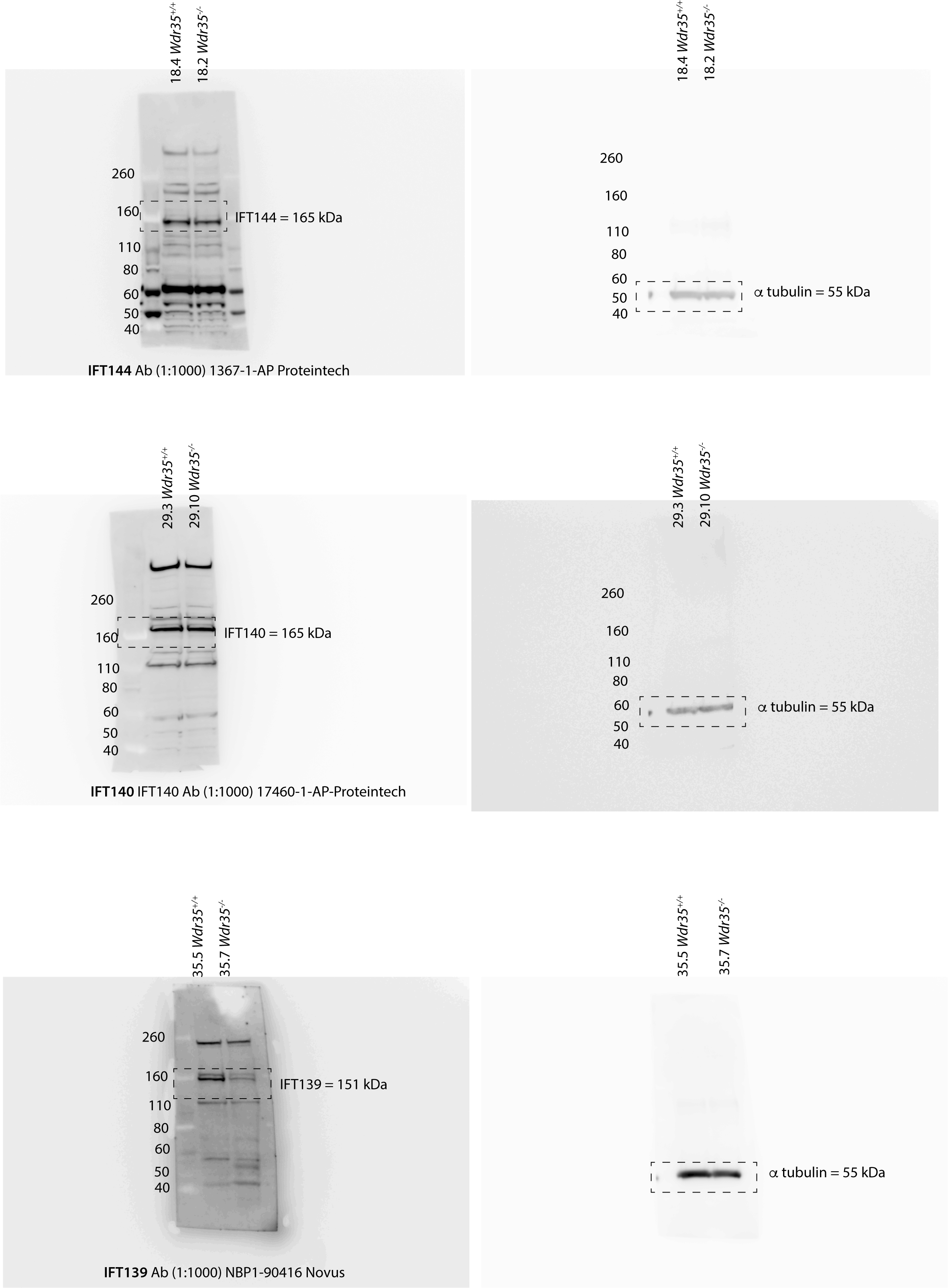

Supplement: Figure 3—source data 1. [file elife-69786-fig3-data1.zip › Figure 3 Source blots upload AUG 2021/Figure3C/Figure 3C Source labelled blots /Figure 3C labelled IFT144 140 139.tif]

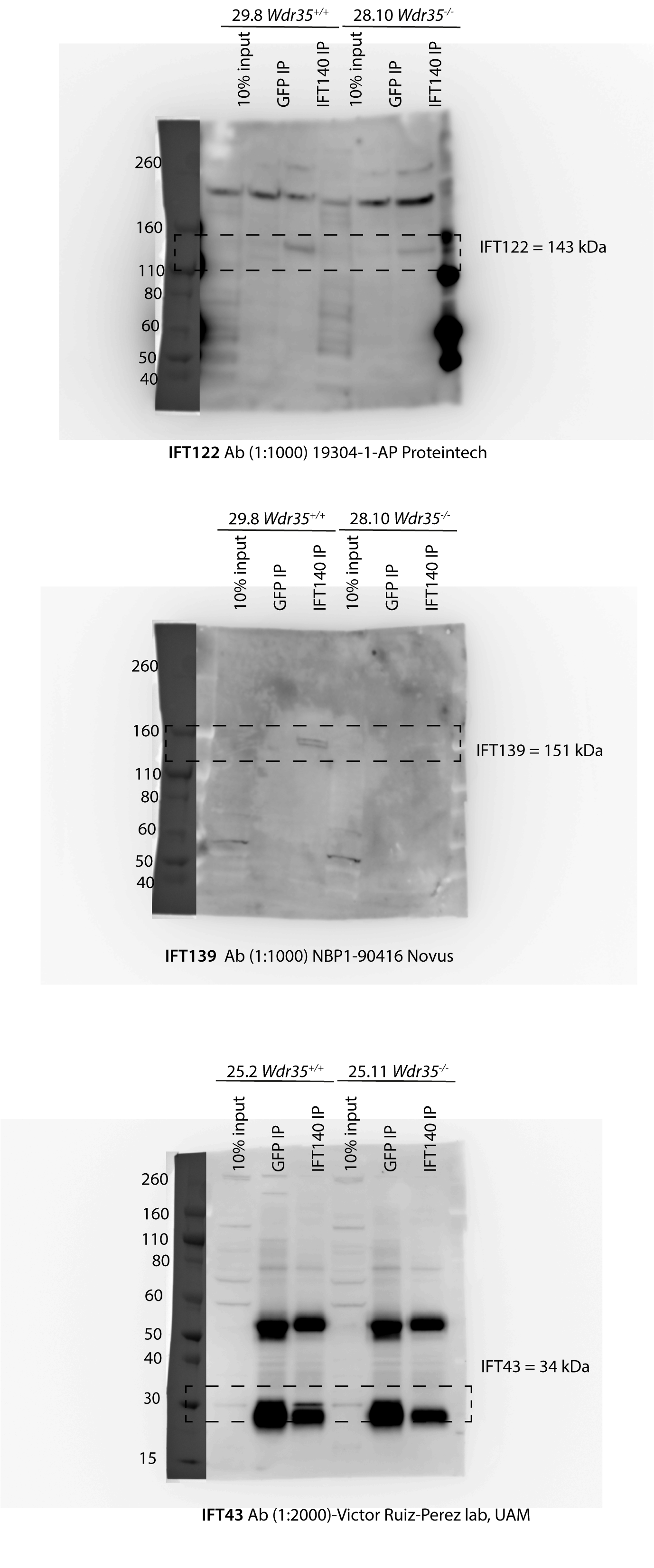

Supplement: Figure 3—source data 1. [file elife-69786-fig3-data1.zip › Figure 3 Source blots upload AUG 2021/Figure3B/Figure 3B labelled source blots /Figure 3B source data labelled 122 139 43.tif]

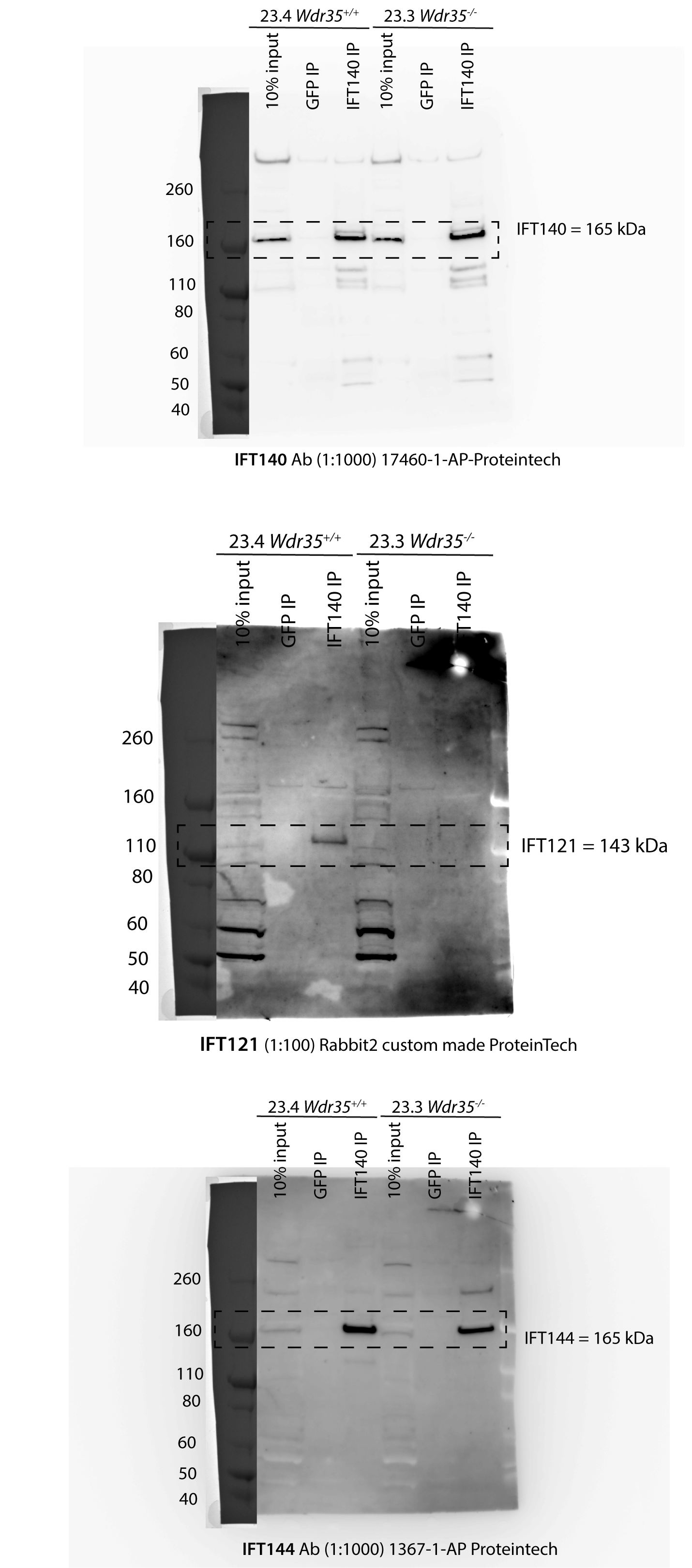

Supplement: Figure 3—source data 1. [file elife-69786-fig3-data1.zip › Figure 3 Source blots upload AUG 2021/Figure3B/Figure 3B labelled source blots /Figure 3B source data labelled IFT140 IFT121 IFT144.tif]

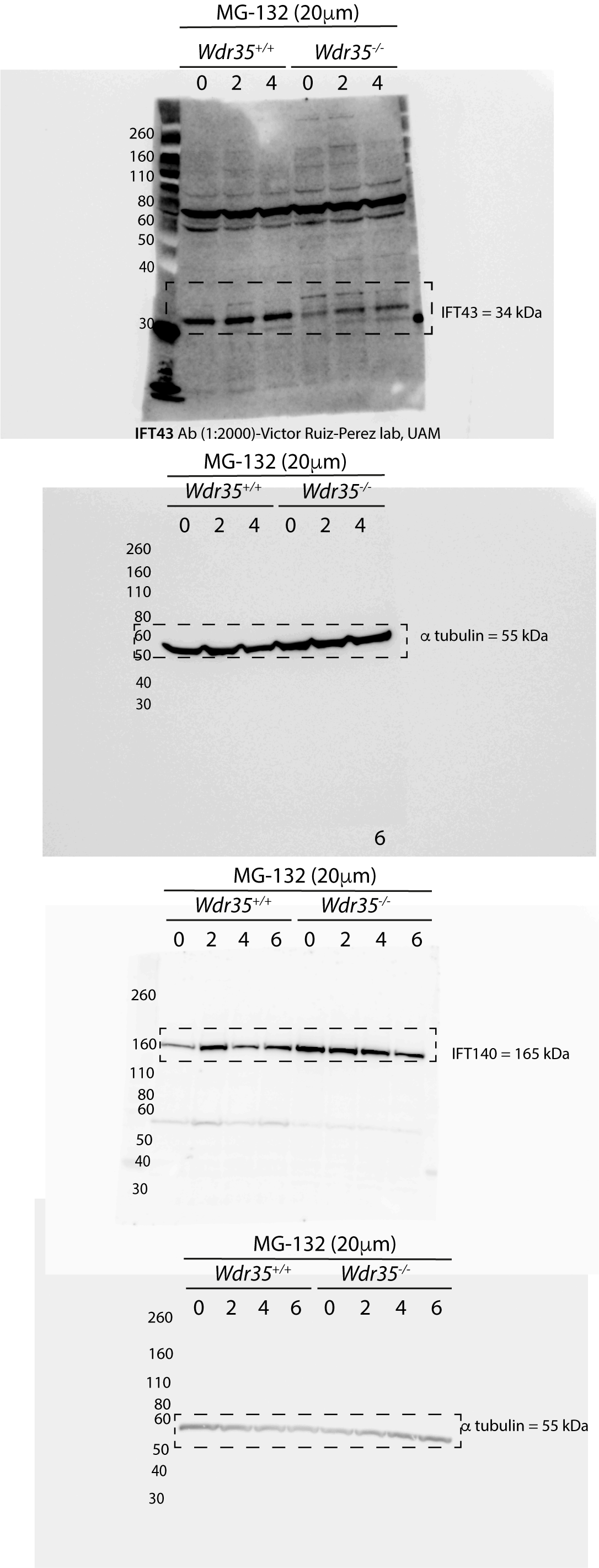

Supplement: Figure 3—source data 1. [file elife-69786-fig3-data1.zip › Figure 3 Source blots upload AUG 2021/Figure3E/Figure 3E labelled source blots/Figure 3E labelled full blots 43 140.tif]

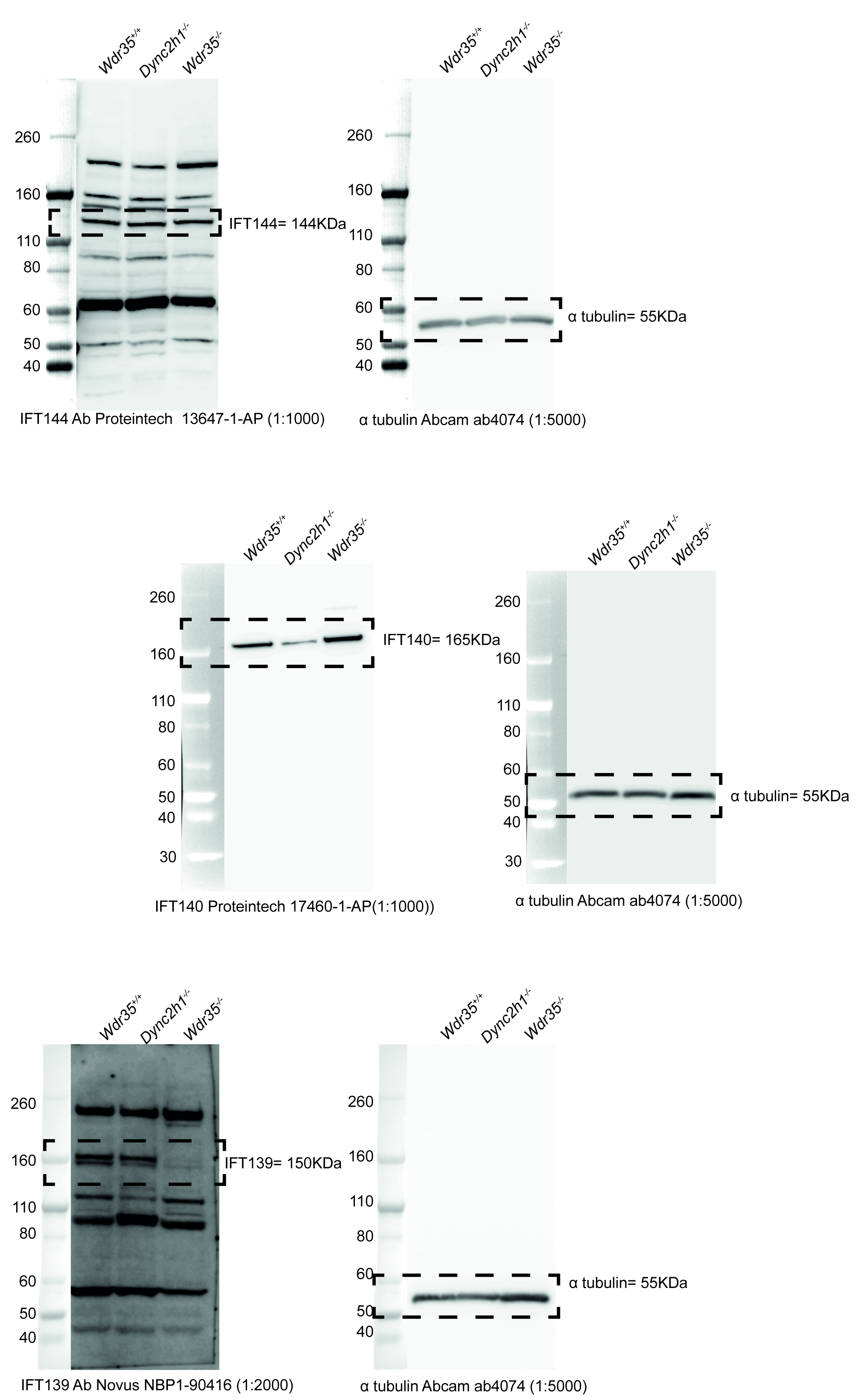

Supplement: Figure 3—figure supplement 1—source data 1. [file elife-69786-fig3-figsupp1-data1.zip › Figure 3- figure supplement 1A and C/Figure 3- figure supplement 1A source blots from MEF lysate/Figure 3- figure supplement 1A labeled source blots from MEF lysate/figure 3 figure supplement 1A labelled IFT144 140 139.tif]

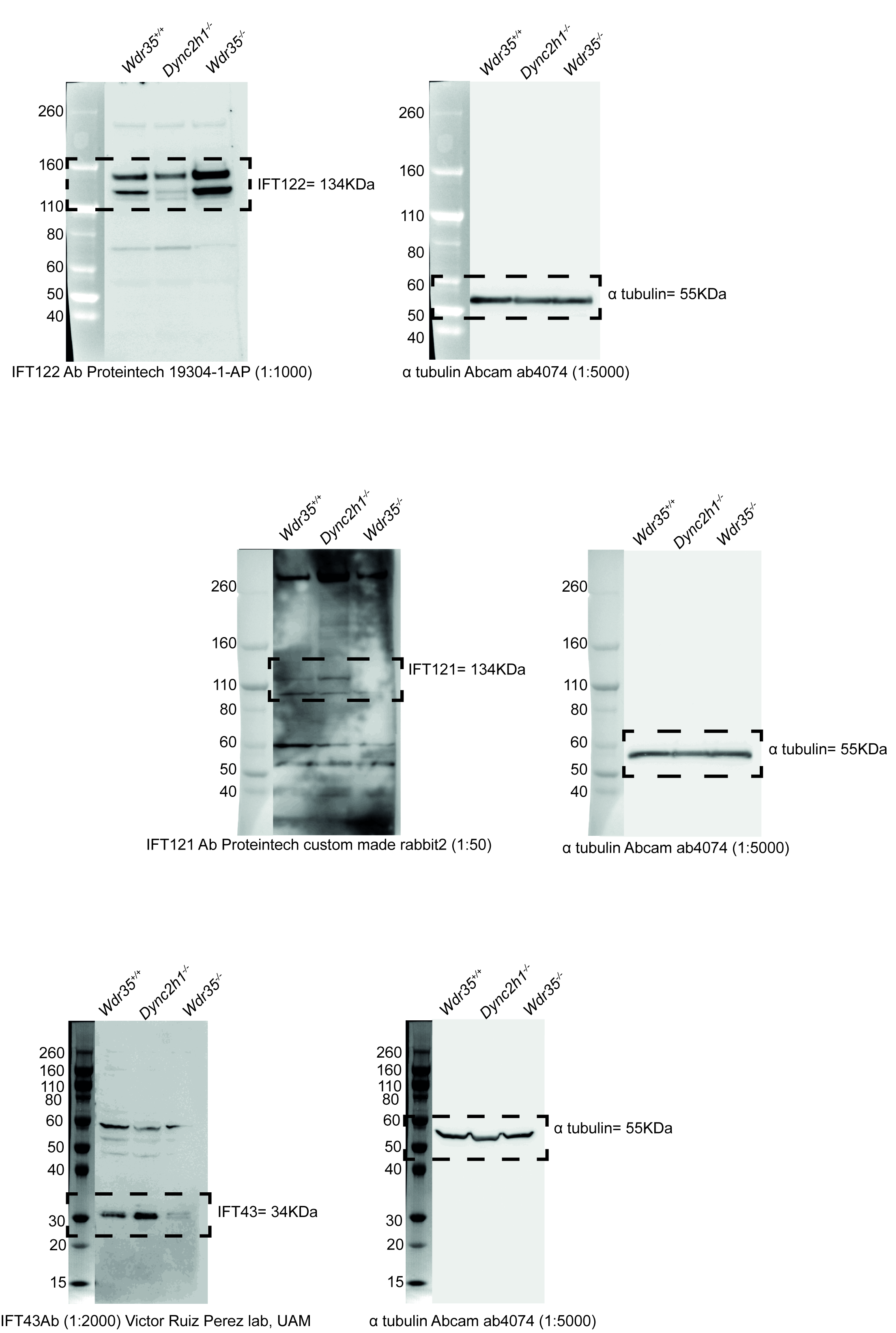

Supplement: Figure 3—figure supplement 1—source data 1. [file elife-69786-fig3-figsupp1-data1.zip › Figure 3- figure supplement 1A and C/Figure 3- figure supplement 1A source blots from MEF lysate/Figure 3- figure supplement 1A labeled source blots from MEF lysate/figure 3 figure supplement 1A labelled IFT122 121 43.tif]

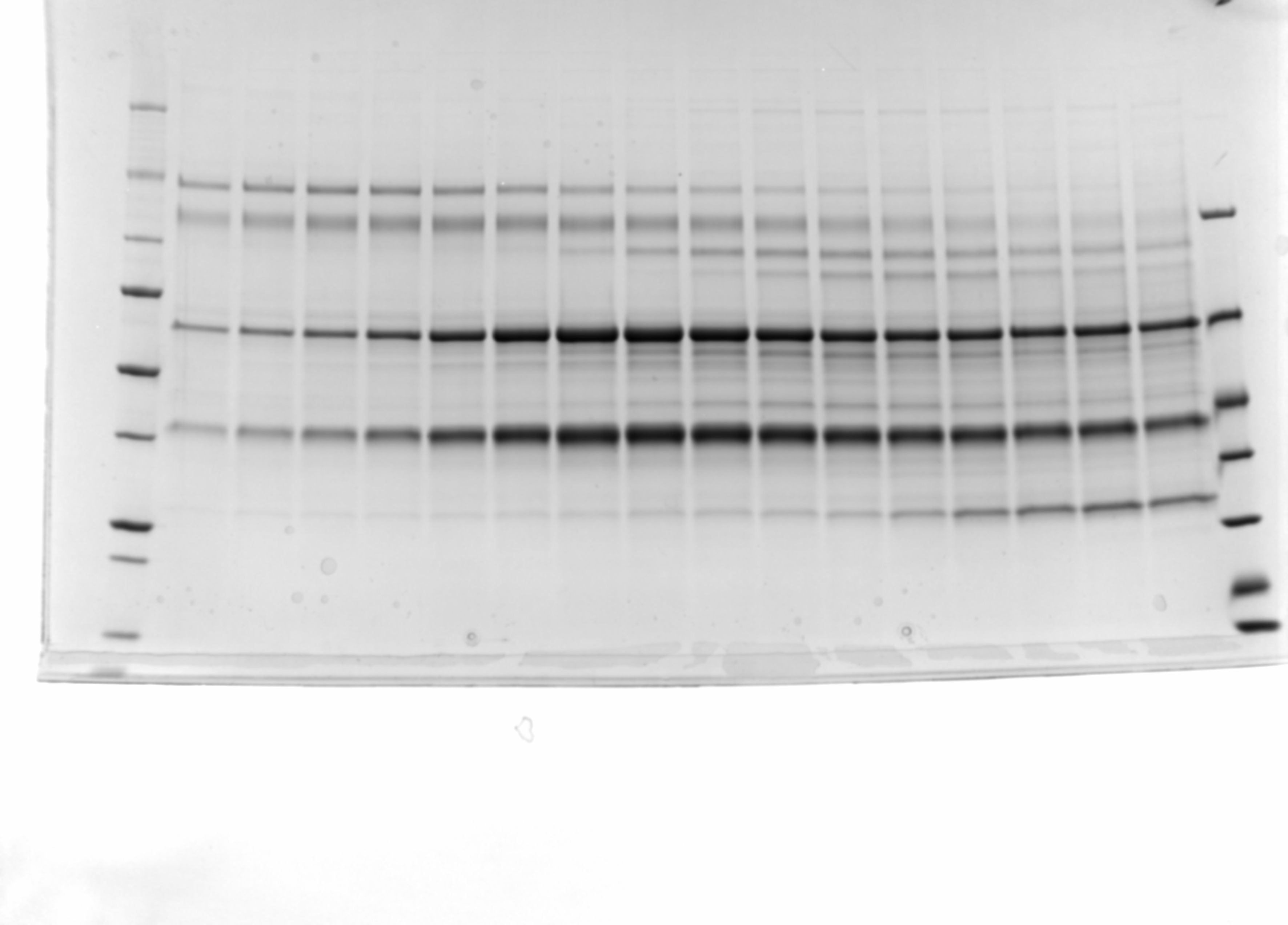

Supplement: Figure 5—source data 1. [file elife-69786-fig5-data1.zip › Figure 5 Source_blots Aug 2021/Figure 5 Source original unlabeled blots/Figure 5-B.tif]

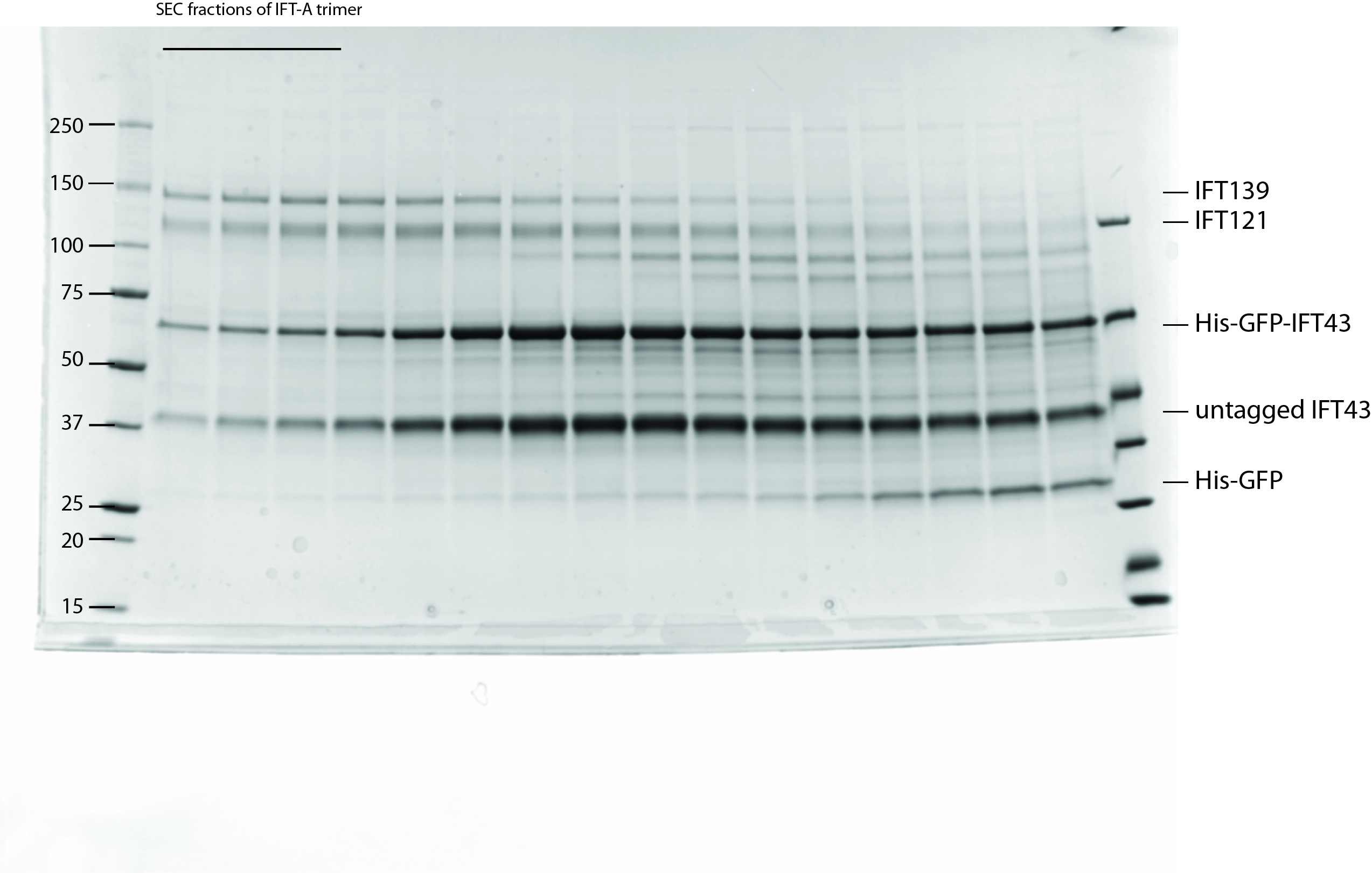

Supplement: Figure 5—source data 1. [file elife-69786-fig5-data1.zip › Figure 5 Source_blots Aug 2021/Figure 5 Source data labelled blots/Figure 5-B.tif]

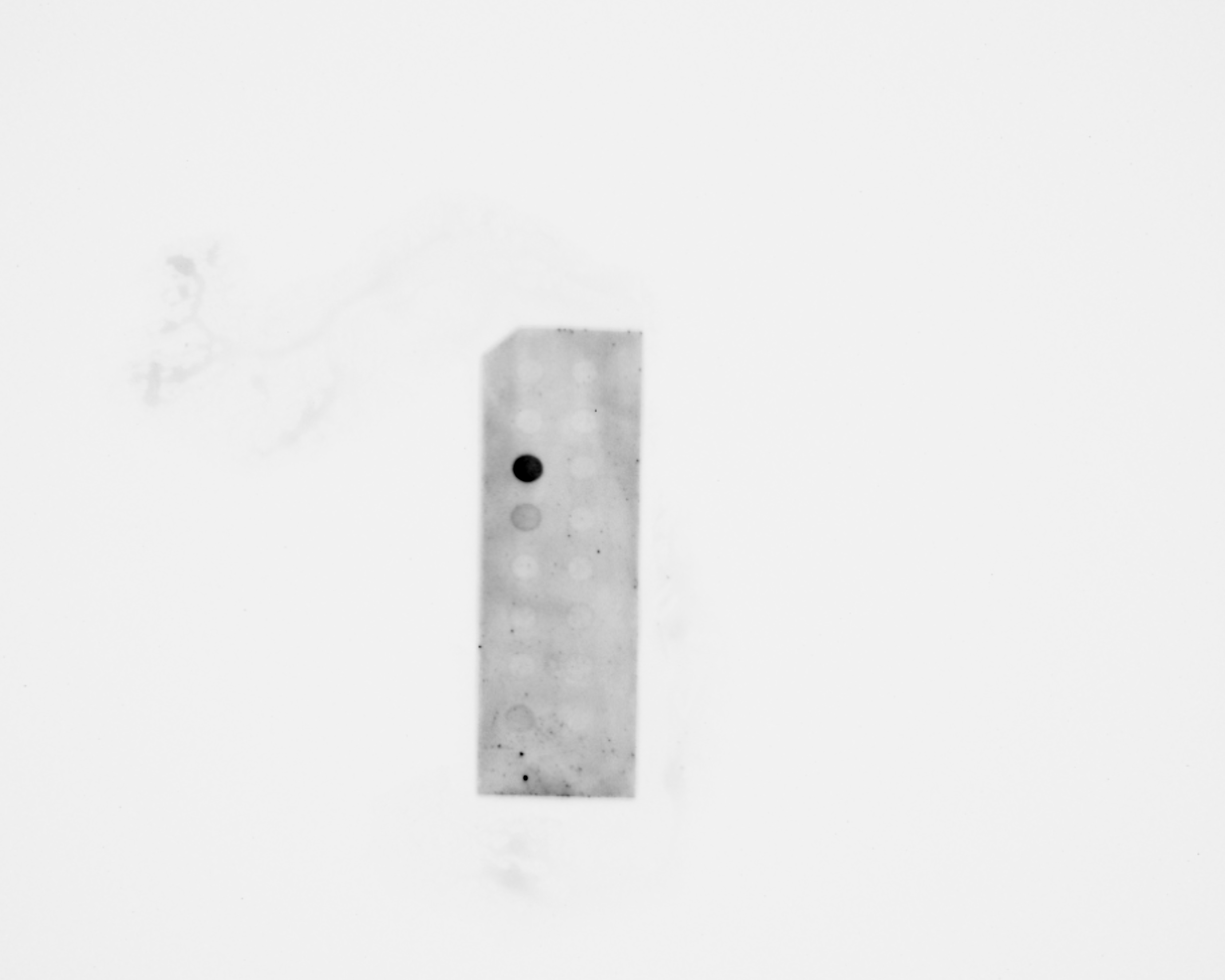

Supplement: Figure 5—source data 1. [file elife-69786-fig5-data1.zip › Figure 5 Source_blots Aug 2021/Figure 5 Source data labelled blots/Figure 5-C.tif]

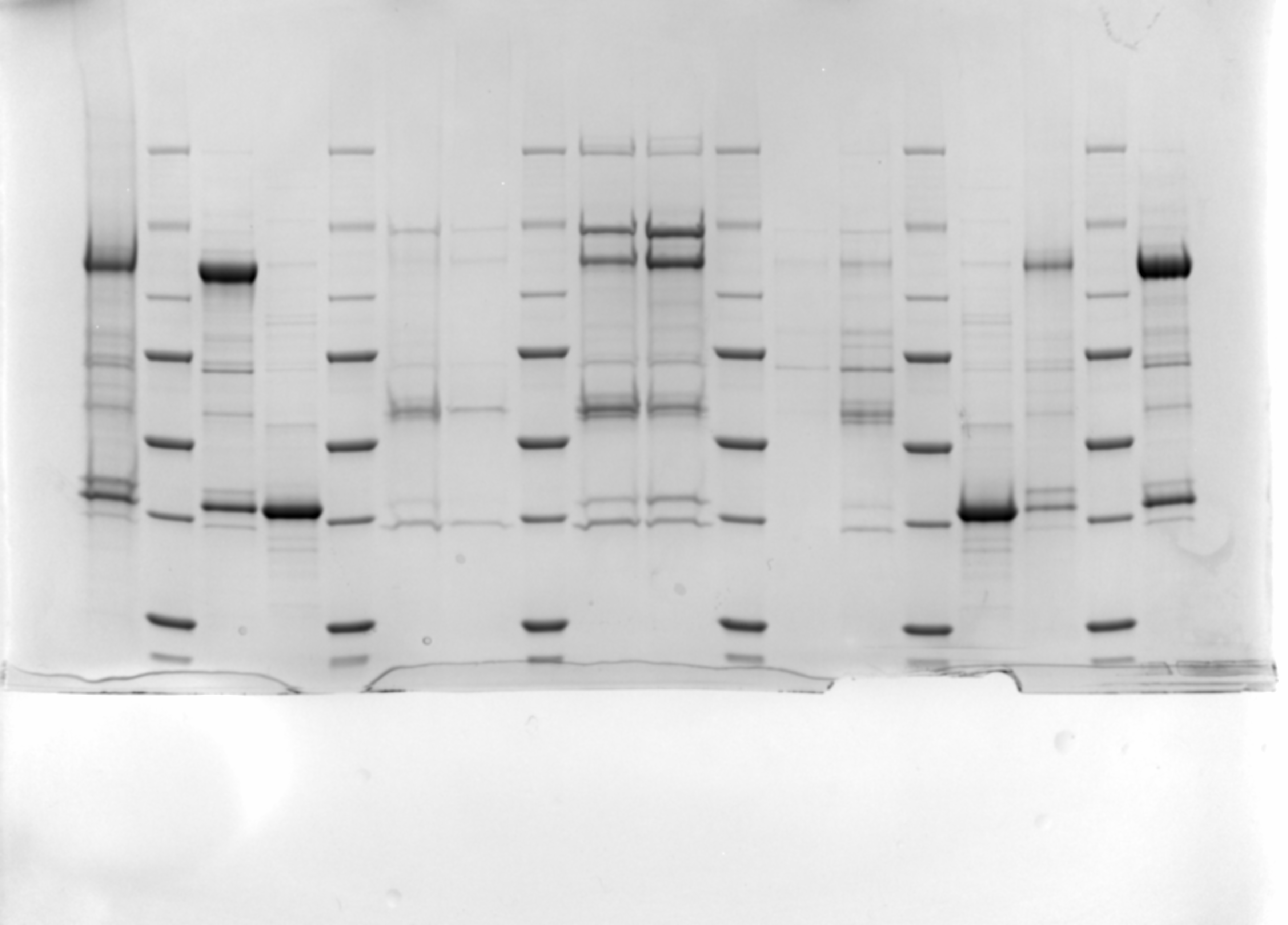

Supplement: Figure 5—figure supplement 1—source data 1. [file elife-69786-fig5-figsupp1-data1.zip › Figure 5- figure supplement 1B-D source data/Figure 5 Source original unlabeled blots/Figure5-Figure supplement 1-B&C (right part).tif]

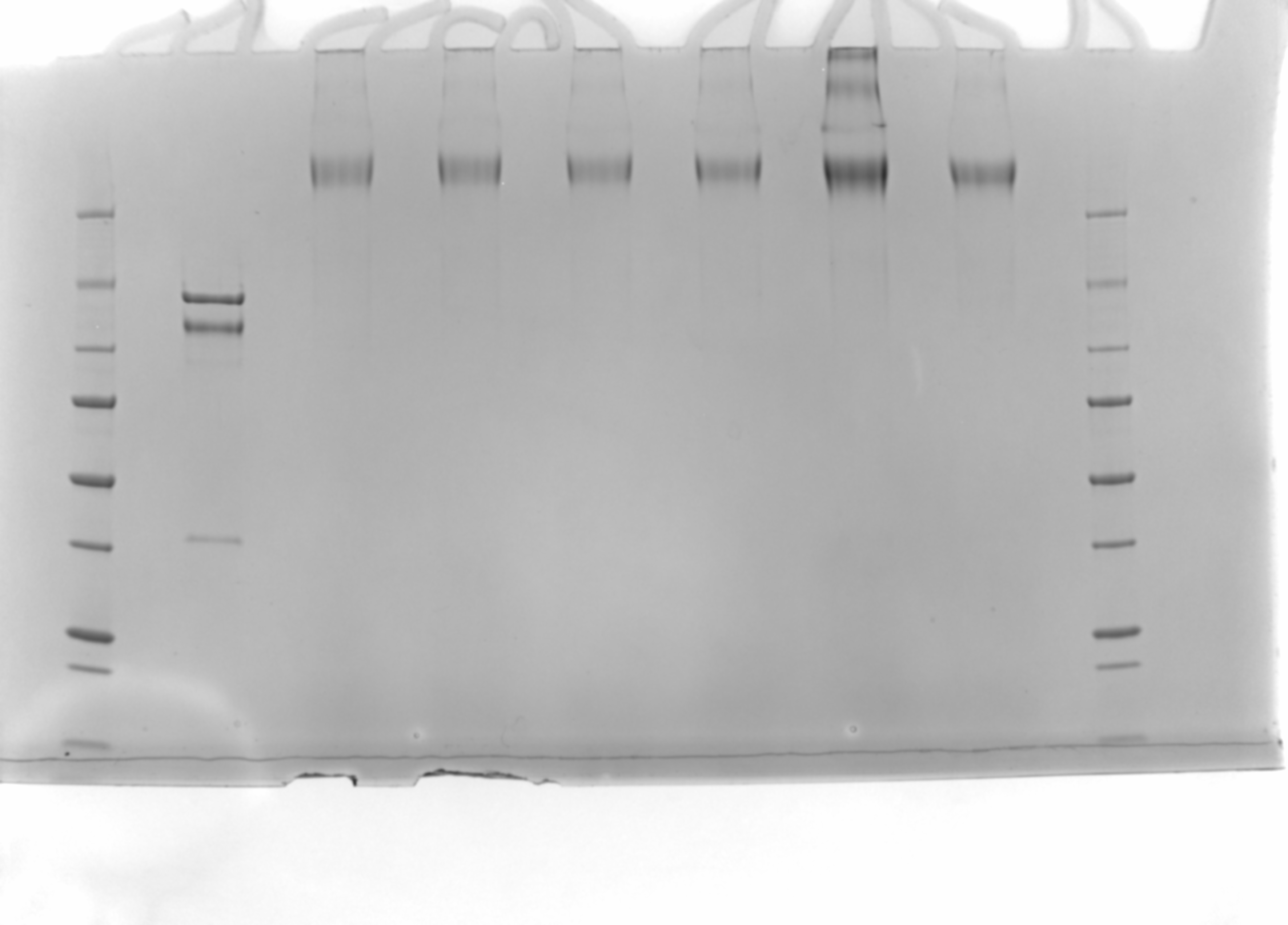

Supplement: Figure 5—figure supplement 1—source data 1. [file elife-69786-fig5-figsupp1-data1.zip › Figure 5- figure supplement 1B-D source data/Figure 5 Source original unlabeled blots/Figure5-Figure supplement 1-D (right part).tif]

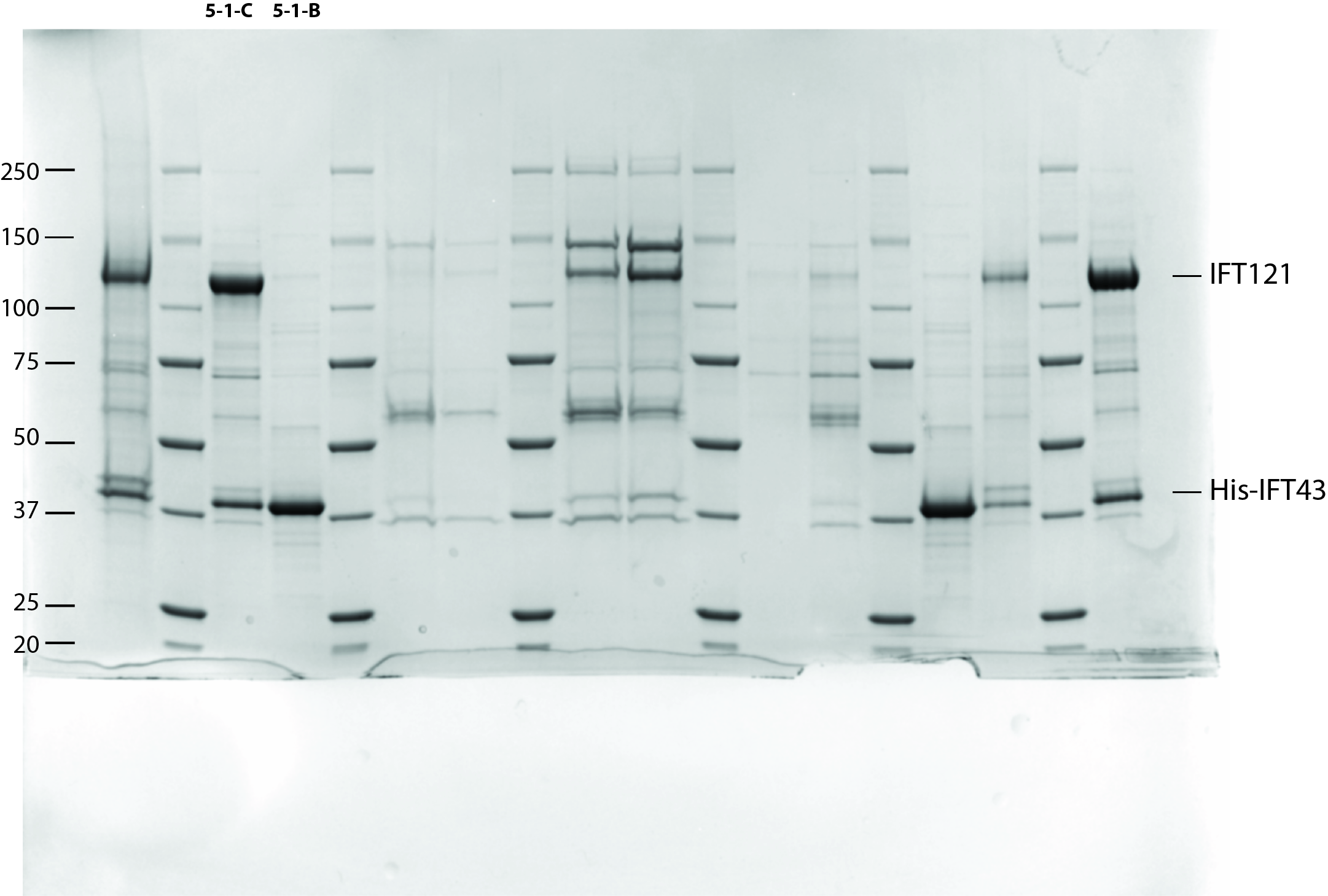

Supplement: Figure 5—figure supplement 1—source data 1. [file elife-69786-fig5-figsupp1-data1.zip › Figure 5- figure supplement 1B-D source data/Figure 5 Source data labelled blots/Figure5-Figure supplement 1-C (right part).tif]

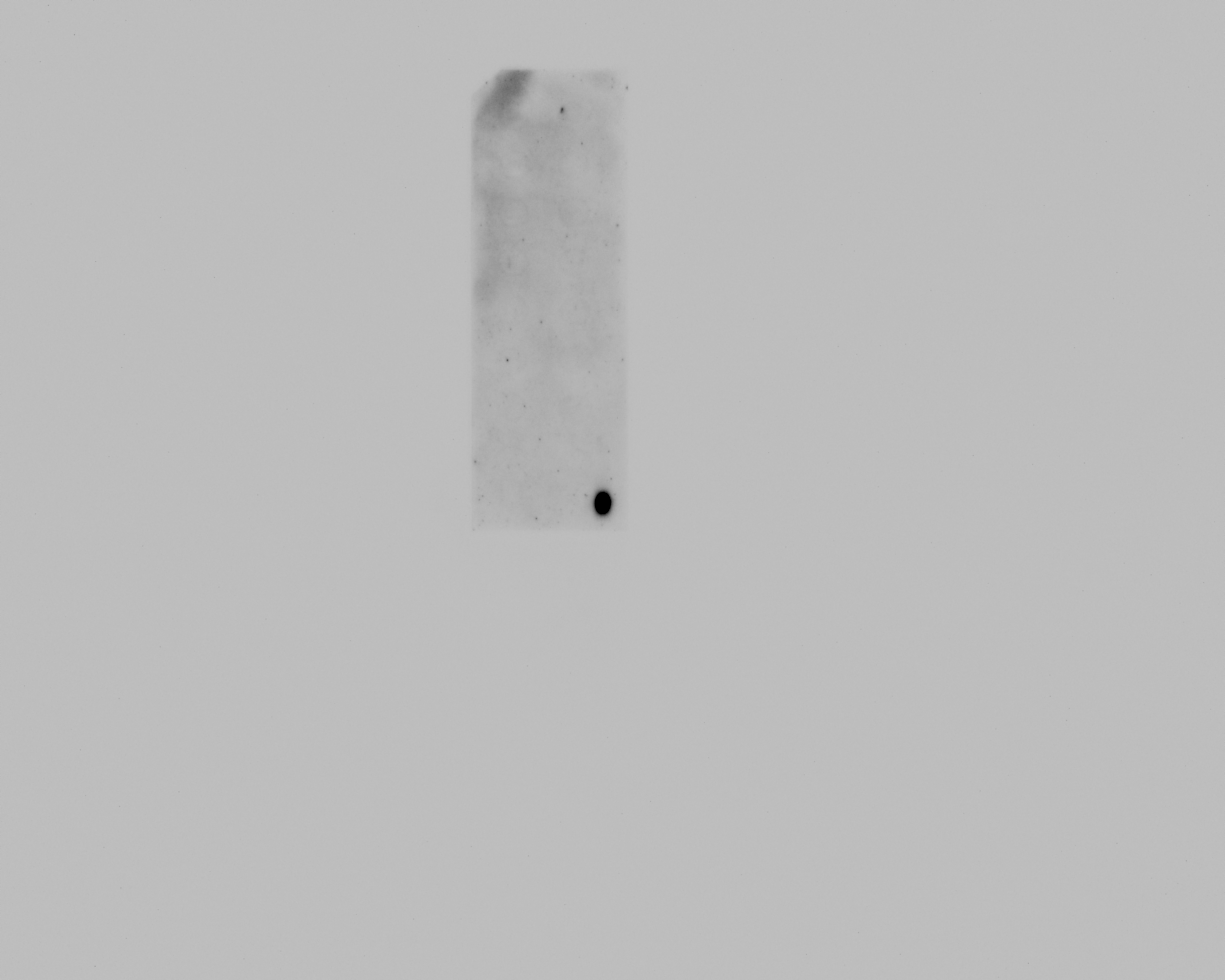

Supplement: Figure 5—figure supplement 1—source data 1. [file elife-69786-fig5-figsupp1-data1.zip › Figure 5- figure supplement 1B-D source data/Figure 5 Source data labelled blots/Figure5-Figure supplement 1-C (left part).tif]

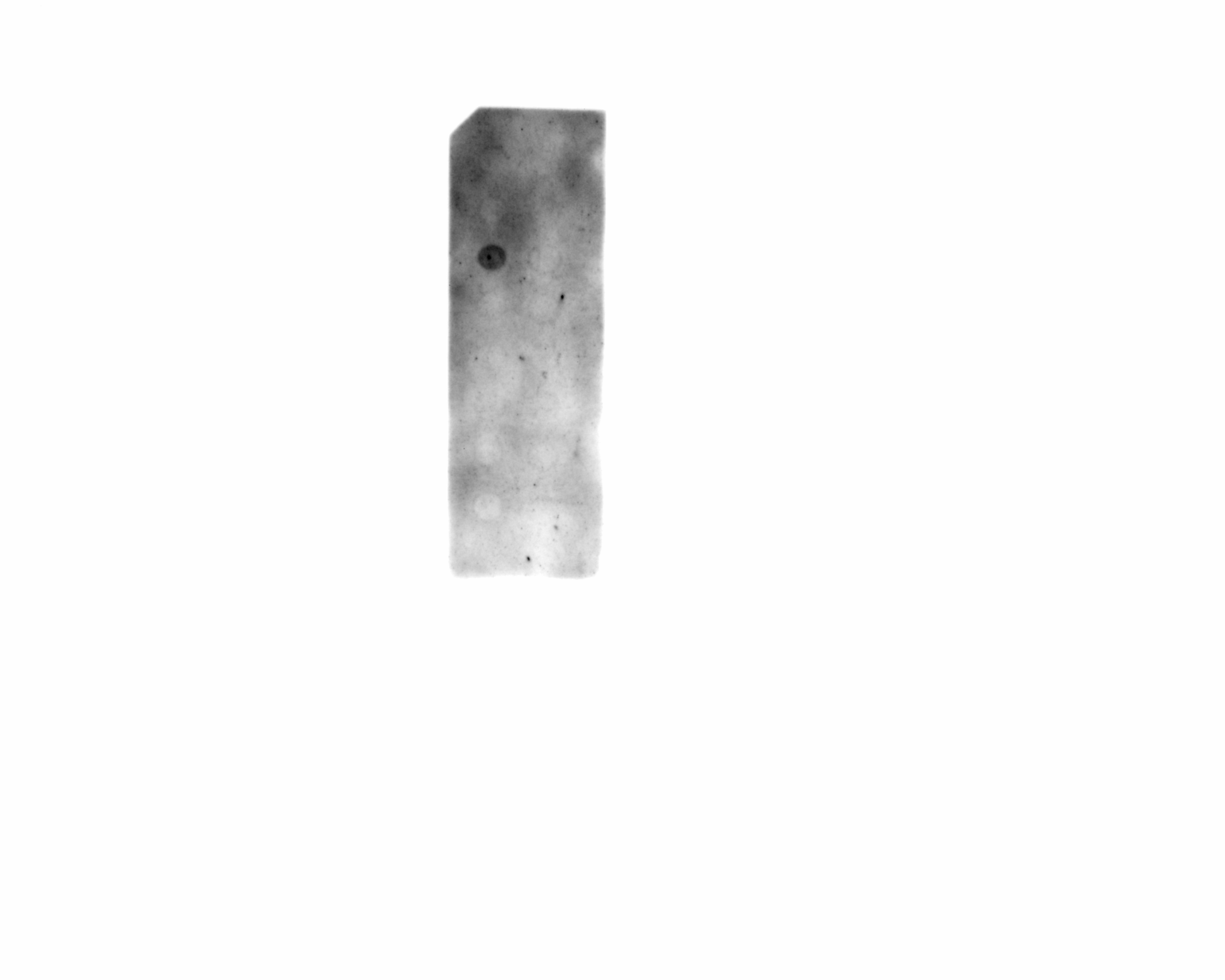

Supplement: Figure 5—figure supplement 1—source data 1. [file elife-69786-fig5-figsupp1-data1.zip › Figure 5- figure supplement 1B-D source data/Figure 5 Source data labelled blots/Figure5-Figure supplement 1-D (left part).tif]

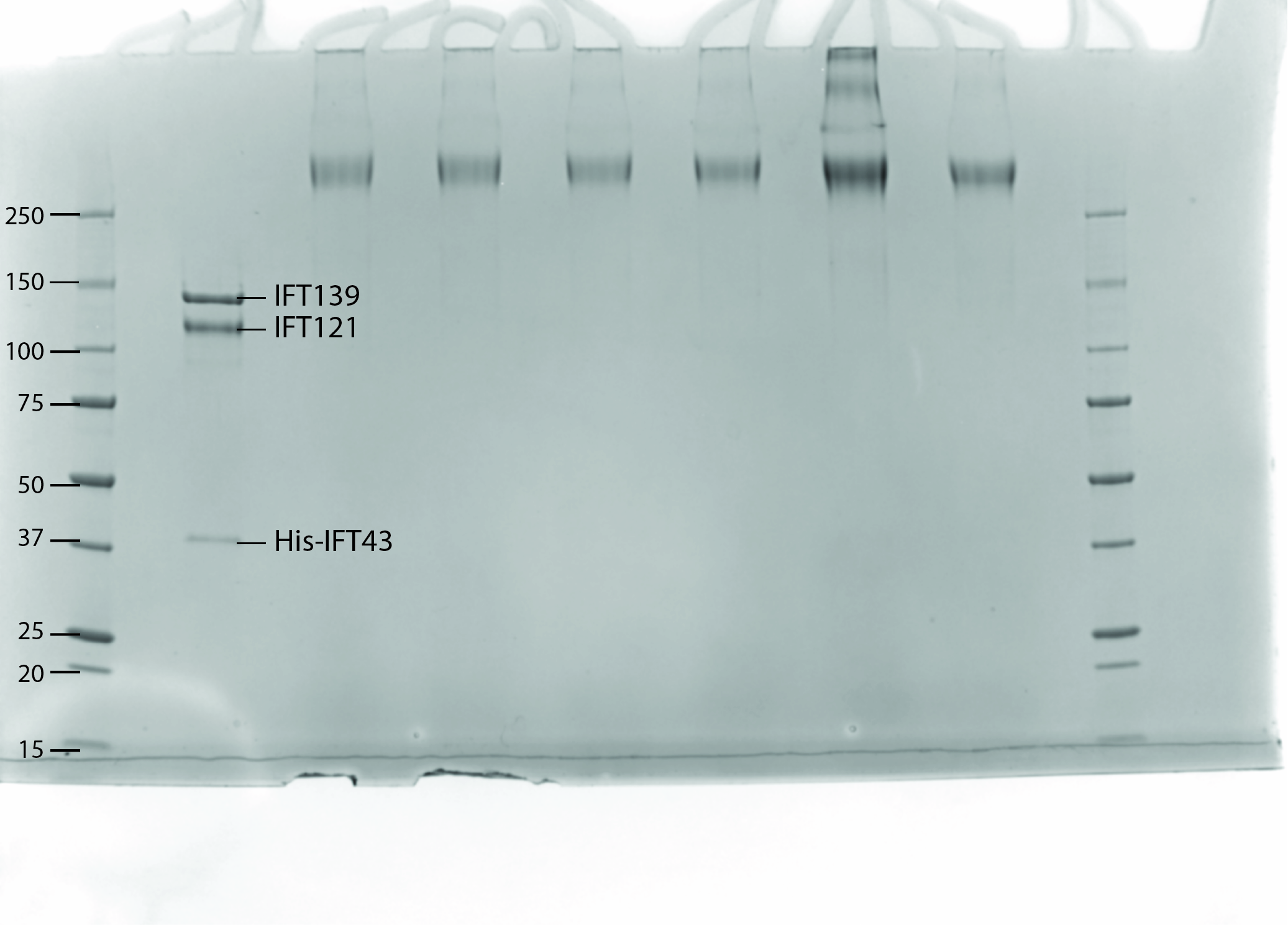

Supplement: Figure 5—figure supplement 1—source data 1. [file elife-69786-fig5-figsupp1-data1.zip › Figure 5- figure supplement 1B-D source data/Figure 5 Source data labelled blots/Figure5-Figure supplement 1-D (right part).tif]

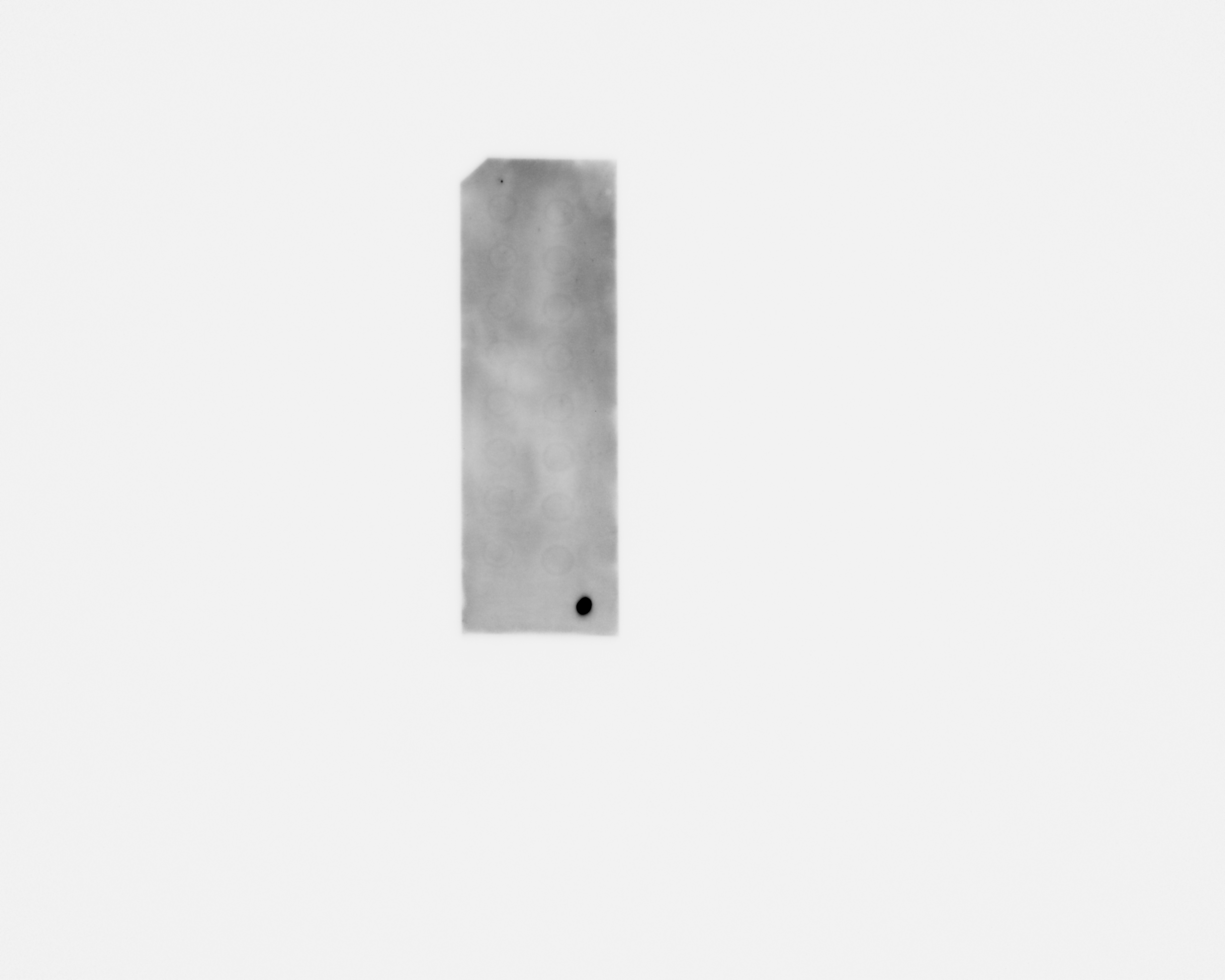

Supplement: Figure 5—figure supplement 1—source data 1. [file elife-69786-fig5-figsupp1-data1.zip › Figure 5- figure supplement 1B-D source data/Figure 5 Source data labelled blots/Figure5-Figure supplement 1-B (left part).tif]
